# Challenges in legitimizing further measures against smoking in jurisdictions with robust infrastructure for tobacco control: how far can the authorities allow themselves to go?

**DOI:** 10.1186/s12954-024-00951-w

**Published:** 2024-02-07

**Authors:** Karl Erik Lund, Gunnar Saebo

**Affiliations:** Department for Alcohol, Tobacco and Drugs, Norwegian Institute of Public Health (NIPH), Folkehelseinstituttet, Postboks 222, 0213 Skøyen, Oslo Norway

**Keywords:** Tobacco harm reduction, Paternalism, Interventions, Tobacco policy, Tobacco prevention, Nicotine, Smoking

## Abstract

**Background:**

According to a recently published study, approximately half of those who currently smoke in Norway have little or no desire to quit despite a hostile regulatory and socio-cultural climate for smoking. On this background, we discuss some challenges that regulators will face in a further tightening of structural measures to curb smoking.

**Main body:**

Central to our discussion is the research literature concerned with the concept of state-paternalism in tobacco control—the line between an ethically justified interference with the freedom of those who smoke and an exaggerated infringement disproportionate to the same people’s right to live as they choose. In countries with an already advanced infrastructure for tobacco control, this dilemma might become quite intrusive for regulators. We ask that if people, who smoke are aware of and have accepted the risks, are willing to pay the price, smoke exclusively in designated areas, and make decisions uninfluenced by persuasive messages from manufacturers—is a further tightening of anti-smoking measures still legitimate? Strengthening of the infrastructure for tobacco control can be seen as a “help” to people who—due to some sort of “decision failure”—continue to smoke against their own will. However, for those who want to continue smoking for reasons that for them appear rational, such measures may appear unwanted, punitive, and coercive. Is it within the rights of regulators to ignore peoples’ self-determination for the sake of their own good? We problematize the “help” argument and discuss the authorities' right to elevate the zero-vision of smoking as universally applicable while at the same time setting up barriers to switching to alternative nicotine products with reduced risk.

**Conclusion:**

We recommend that a further intensification of smoking control in countries that already have a well-developed policy in this area requires that regulators start to exploit the opportunity that lies in the ongoing diversification of the recreational nicotine market.

## Background

The point of departure in our commentary is a recent study indicating that half of those who currently smoke in Norway have little or no desire to quit despite a hostile regulatory and socio-cultural climate for smoking [1]. On this background, we discuss some challenges that regulators in jurisdictions with a robust infrastructure for tobacco control will face in a further tightening of structural measures to curb smoking. Strengthening of the infrastructure for tobacco control can be seen as a “help” to people who—due to some sort of “decision failure”—continue to smoke against their own will. However, for those who want to continue smoking for reasons that for them appear rational, such measures may appear unwanted, punitive, and coercive. Is it within the rights of regulators to ignore peoples’ self-determination for the sake of their own good?

We problematize the “help” argument and discuss the authorities' right to elevate the zero-vision of smoking as universally applicable while at the same time setting up barriers to switching to alternative nicotine products with reduced risk. Central to our discussion is the research literature concerned with the concept of state-paternalism in tobacco control—the line between an ethically justified interference with the freedom of those who smoke and an exaggerated infringement disproportionate to the same people’s right to live as they choose.

## Main text

### How many people who smoke want to quit?

When representatives of the tobacco control community argue for tax hikes, reduced accessibility to cigarettes and limitations on opportunities to smoke, it is pointed out that a large segment of those who smoke actually want to quit, and that putting up constraints may help them pursue their desire [2]. Typically, estimates have been that approximately 70% of those who smoke are interested in quitting [3–5]. However, these estimates seem to emerge from selective use of one-item indicators of quitting interest, often in combination with a procedure where binary response categories (yes/no) are utilized or where respondents with mid-scale values on Likert-scales for quitting interest are included in the group defined as people with a desire to quit smoking.

A more robust and valid measure for quitting interest is to use multiple indicators to build an index and then use the index distribution to define the fraction of those who smoke with and without interest in quitting smoking [6]. In a paper recently published in the Journal of Smoking Cessation, we examined how a sample of Norwegians who smoked were distributed on four indicators of interest in quitting: (i) expressed degree of desire to quit, (ii) prediction of future smoking status, (iii) reported plan for quitting smoking, and (iv) statements on previous quit attempts [1]. Based on these items, an index was constructed, and the respondents’ scores were distributed on a 5-point rating scale from very low (1) to very high (5). Nearly half—48%—were classified as having very low (24%) or low (24%) interest in quitting smoking—in the literature often labeled “consonant smokers”^1^ [7]. One-fifth—20%—were categorized with an average interest in quitting. And only one-third—33%—had very high (13%) or high (20%) interest in quitting—labeled “dissonant smokers” (see [1] for a discussion on misclassification on the index). For the half with a desire to keep on smoking—the consonant smokers—the help argument may not carry much merit.

### Use and misuse of the help argument

The help argument to justify stricter smoking regulations is based on the idea that nicotine addiction affects the ability to choose in such a way that the decision whether to light another cigarette is disturbed by signal-controlled ignition reactions (cues and cravings) [8, 9]. Irresistibility and overwhelming desire can cause people who smoke to act contrary to their own interests and conviction. In theories of rational action, weakness of the will is considered the antithesis of rational behavior [10]. For actors who smoke contrary to their volition due to some kind of decision lapse, tightening of the structural constraints for smoking might indeed provide opportunities to reintroduce self-regulation and to comply with their real wishes [11–14]. These people might find constraints on their choice acceptable because it may go into a strategy for executing self-control and help them establish a precommitment to be locked in a more credible behavior in the future [15]—a type of self-binding [9].

However, in invoking the help argument, the authorities must demonstrate that i) the decision failures will result in serious consequences for those who smoke and, moreover, ii) that a sizable segment of the group that smoke in fact have a desire to quit [16].

Failure to pursue a desire to quit smoking can undoubtedly have *serious* consequences for the future health status [17], become an economic burden [18] and inflict social stigma [19–23]. However, how people who smoke are distributed on a measure of interest in quitting will, as emphasized at the outset, be dependent on the questions we choose to elicit information from. From an authentication perspective, it will make a big difference if the percentage of those who smoke against their own will is approximately 30% as suggested by Saebo & Lund [1], or 70% or beyond as often claimed by tobacco control representatives [24, 25].

Even though intensification of structural measures is based on a charity principle and a care ideology, it may entail that those who wish to continue smoking will face increasing difficulty, loss of welfare, loss of autonomy, and social disqualification [26, 27]. Studies have revealed that proposed restrictions on purchase availability, reduced access to outdoor smoking, and tax hikes on cigarettes have little support among people who currently smoke [28, 29]. As smoking in most western countries disproportionately affects people in lower socio-economic groups, ethnic minorities, and marginalized groups often with high incidence of mental disorders [30, 31], already vulnerable populations will thereby be hit the hardest [32–34].^2^

When arguing for stronger tobacco control measures, regulators should perhaps try to avoid the help argument from becoming a substitute pretext to regulate unwanted behavior in a situation where intensification lacks the support of the group they are intending to help. On the other hand, the point could also be made that those who smoke do not realize that these measures will help them, but that they will nevertheless be grateful afterward.^3^

### The right to define the self-interests of people who smoke

Authorities introduce structural measures—physical, economic, and legal constraints—so that people who smoke will act in accordance with what the authorities believe is in the smokers’ actual enlightened self-interest [37, 38]. When the authorities exercise the right to decide what is best for their fellow citizens, it means that the authorities elevate their value to a universal matter of common cause [39, 40]. Intensified use of structural measures to curb smoking is built on a moral foundation that is difficult to oppose—they save lives. As a value, it could be argued that health takes precedence over competing values, e.g. the value of immediate pleasure from smoking a cigarette. In effect, health is a prerequisite to positive liberty, worthy of special moral importance and legal respect [41]. Therefore, health may not simply appear as any another good, but one necessary to enable individuals to exercise their other liberties or attain their personal goals—a “meta-capability” [42].

However, also the value of health belongs to a normative domain without necessarily having any priority over other values that can also provide welfare to consumers. Wilson [43] states that we stand in need of an account of how important health is *relative* to the importance of other goods that a just society should be trying to secure for its citizens. As it appears from the Saebo & Lund study [1], far from all of those who smoke seem to regard future health status as a higher welfare value than the present pleasure they derive from continued smoking. This might in part be written off as a type of myopic behavior where these individuals have difficulties in discounting the long-term effects of their current actions [44], which in that case may legitimize interventions from the state. However, according to Skog [45], most people who smoke have conflicting motives but actually make calculated and rational considerations. When they choose to continue smoking, Skog argues it is simply because they conclude that smoking is so stimulating that it exceeds a health-related desire to quit.

After 60 years of tobacco control, the authorities have ensured that most of the people who smoke nowadays in countries with a long-lasting anti-smoking policy are well informed about the health risks [46, 47], although their understanding of what causes the harm might be inadequate [48]. One can plausibly argue that smoking in these countries is carried out by educated persons who have chosen to accept the health risks. Moreover, authorities tax cigarettes to cover most—if not all—the externalities inflicted from smoking injuries [49]. In addition, smoking has been restricted to designated areas and most communication channels from manufacturer to consumers have been eliminated. In the case of Norway; an ad ban since 1975, a display ban since 2008 and plain packaging since 2018. In fact, Norway is ranked among the top-five European countries as regard robustness of tobacco control [50].

As a result, it can be argued that people who smoke today in western countries have given an ‘informed consent’—according to a decision in the Norwegian High Court[51].^4^ Furthermore, those who smoke probably pay their way at the level of excise taxes on cigarettes [52, 53] and they tend to comply with regulations and carry out their activity in designated areas without harming others [54]. Lastly, consumers make their decisions about smoking in an epistemological climate unaffected by persuasive messages from the manufacturers. A timely question is therefore that if consumers are informed and consonant, willing to pay the cost, act regulatory-compliant, appear unexposed to seductive marketing *and* possess a desire to continue smoking, do the regulators then have the right to tighten the measures further and in doing so, ignoring these people’ self-determination for the sake of their own good?

Intensification of structural measures have no doubt been important both for increasing the rate of quitting smoking among “established smokers” and for reducing incidence of smoking among the youth [55]. As in most other countries [56], consideration for the youth has been especially important when designing the Norwegian tobacco policy [57], as well as policies in most other western countries in an advanced stage of the smoking epidemic. Even if people who smoke are aware of the risks and should pay their way, policymakers still wish to raise cigarette taxes to reduce the number of adolescents taking up smoking.

However, in Norway, the prevalence of daily smoking among the young people has declined to 1–2% [57]. In a situation where cigarettes no longer seem to be part of the youth culture, it might perhaps be timely to pay more attention to the welfare of the people who smoke? An appeal to switch to less dangerous nicotine products could, for example, be conveyed on inserts in cigarette packs. This message channel targets people who smoke and will leave everyone else unexposed. As we will elaborate toward the end of the paper, it may appear somewhat easier for regulators to legitimize intensified use of structural measures to curb smoking if they simultaneously facilitate a product transition [38, 58, 59].

### From soft to hard paternalism in tobacco control

Discussions of autonomy and paternalism have for a long time been at the forefront of contemporary public health ethics [60]. Balancing intensification of structural measures against individual freedom has also been a recurring theme in connection with the development of steadily stronger infrastructure for tobacco control [38, 61, 62]. Some ethicists’ opinion that pro-paternalism in tobacco control will be appropriate to achieve a, for them, desired gradual transition toward a blanket prohibition on the sale of cigarettes [2,12,13,0.63,64]. Some claim that critical reflections upon the limits of state interventions to curb smoking could be staged by the tobacco industry to prevent market interference [65, 66]. On the other side, the most vocal critics of paternalism belong to economic liberalists [67–69], typically citing the anti-paternalistic stance of John Stewart Mill [70] that a government is only justified in interfering with individual liberty to prevent harm to others. Patronage rhetoric about the Nanny State is common among these authors.

Most lexical definitions of state-paternalism emphasize the interference of authorities against some one’s will motivated by a claim that the persons interfered with will be better off or protected from harm [71]. Placing risk-information on packs, introducing a reasonable level of excise tax, putting up barriers for glorifying messages about cigarettes, and even restricting smoking to certain areas can be interpreted as well-meaning guidance to our choices and could be defined as soft paternalism [72]. These measures are certainly freedom-restricting but only in a weak sense because they simply inform and ‘nudge’ consumers while leaving the final decision up to themselves [14]. Soft paternalism aims to influence behavior by operating on a person’s desires from the inside [64].

In comparison, hard paternalism can be understood as restricting a competent adults’ liberty for their own good under conditions that violate their autonomy in a more intrusive way. Instead of nudging in a welfare-maximizing direction, hard paternalism also deprives people of the option of choice—the opportunity set [73]. This amounts to a violation of basic ethical values and implies that hard paternalism requires extensive justification and usually is much more controversial than soft paternalism. Examples of hard (or ‘coercive’) paternalistic interventions in current tobacco control are extensions of smoking bans onto outdoor public places and banning sales of tobacco to individuals born after a certain year (e.g. 2010). In smoking control, paternalism raises questions concerning regulators’ legitimacy; what are, in fact, the reasonable limits of state intervention in the lives of those who smoke? Regulators must determine the line between an ethically justified interference with the freedom of individuals who smoke and the exaggerated infringement of their freedom, disproportionate to their right to live as they choose.

Scholars have provided explicit and reasoned approaches to conducting an ethical analysis of paternalistic public health policies [1, 74], and a tobacco-specific analysis of a paternalistic justification has been conducted in the case of plain packaging [75]. However, these analyses are often conducted on a case-by-case basis. Ethical analysis that addresses the entire concerted package of smoking preventive measures are scarce. Moreover, ethics is neither the only factor, nor probably the most decisive one, to be considered when regulators decide whether to implement an intervention. Typically, decision-makers take a more colloquial approach without guidance from such types of analytical frameworks [76], and they design policy interventions in accordance with what they believe will resonate with the public (especially the voters) at any given time. Consequently, the policy might then become vulnerable to fluctuating emotional moods in the population, changes in norms and not least to the social and demographic characteristics of the group affected—the people who smoke. Persons who smoke in the Nordic countries—and in other countries in the endgame-phase of the cigarette epidemic—have become a decimated and socially declassified group without influential spokespersons in the corridors of power. Thus, they appear as easy ‘push-overs’ for politicians who often portray their efforts to curb smoking as a crusade against the evils of the tobacco industry.

By contrast, the organized adherents of stricter tobacco policy tend to be upper middle class, occupy important positions in society and are schooled in the code of contact with the authorities [77, 78]. Officials who craft policy make decisions for people who are very different from them. However, the skewed distribution of social, cultural, and economic capital between the unaffected senders of proposals and the affected recipients is rarely considered to be a problem when the tobacco policy is formulated [38, 79]. To justify hard “coercive” paternalism, one must argue that officials are better judges of what will promote a person’s well-being than the person who is subject to coercion, and that their judgment should be granted authority in the law. People who smoke do indeed suffer from cognitive biases, but also public officials may have their own biases to contend with. For example, they may be overly optimistic that their choice to coerce those who smoke will not be harmful on balance, which may cause them to enforce policies that disproportionately burden a marginalized and stigmatized group and display low tolerance for a risky behavior [38].

Studies of tobacco policy in the Nordic countries have claimed that the states now stand for an overall hard paternalistic line [80–82]. The governing authorities define not only which means benefit society as a whole, but also which goal is desirable—namely, a tobacco-free society. In the case of Norway, Saebo [83] claims that the views of those who smoke have gradually become absent in the debate on structural measures, and that their perspectives and experiences hardly are discussed in today's tobacco policy strategy plans and consultation notes. He proposes to take into account this group based on the idea of democratic representation and points out that user participation has become more common when regulators design policy for other drugs taking into account self-understanding and dignity of the people who consume. Also from a social justice perspective, it may be argued that the state should recognize the views of the people who use in the fight against tobacco-related disease.

### Paternalism and addiction

Nicotine is the dependency forming ingredient in cigarettes. In a report from the U.S. Surgeon General in 1988, nicotine was categorized with an ‘addictive’ potential compared to heroin or cocaine [84]. This might perhaps be true for nicotine uptake from smoking, but scholars emphasize that the dependency potential from non-combustible nicotine products is lower [85, 86]. Most lexical definitions of addictions emphasize that compulsive use will result in ‘serious net harm’. Under this definition, pharmaceutical nicotine products approved for use in smoking cessation would not meet the threshold for classification as an addiction. Whether non-combustible nicotine products for recreational use will qualify might also be a topic for further debate. There are residual uncertainties about the risk of these products but compared to smoking the risks probably differ fundamentally rather than incrementally.

The prevailing opinion has been that a majority are ‘addicted to nicotine’. A central dispute is whether smoking brings about a change in the person that in effect will deprive the persons capacity to choose. And because the autonomy of people who smoke might be compromised by nicotine dependency, there might be a justifiable case for hard paternalism [12, 63, 64].

But how large is the segment of those who can be defined as dependent? Conceptualization and measurement of ‘nicotine addiction’ has been extensively discussed. Numerous measures have been put forward that differ in their theoretical underpinnings, whether they are dichotomous or continuous, and whether they are single or multi-dimensional [87]. Estimates will vary according to definitions and the data source applied.

If we apply a definition which means that the consumer must meet three conditions—i) regular use (daily), ii) subjective recognition of potential harm from own consumption and iii) persistent use despite a strong desire to stop using (so-called akrasia) [45, 88]—only 15% of those who currently smoke in Norway can be characterized as addicted. This estimate is based on the fact that around 50% of the Norwegians who smoke use cigarettes only occasionally (contextually conditioned use). And in the remaining half who smoke daily, approximately 1/3 fall in the category 'continues against their own will' on the Saebo-Lund index for interest in quitting [1]. Other studies also indicate that a substantial number of people who smoke do not meet criteria for nicotine addiction [89–92].

Whether addiction in itself is a legitimate justification for paternalistic state interventions, and in that case what proportion of smokers that must be assigned this characteristic to trigger an intervention, will be a matter for further debate. The view that people who smoke lose control over their cigarette consumption may remain popular in some circles of the tobacco control community, because some parties have strong vested interest in sustaining it. The medical establishment gets strong benefits regarding persons who smoke as out of control, because it means that people will need professional help. Supporters of paternalistic interventions toward nicotine use can gain greater legitimacy for their view if the prevailing opinion is that the behavior is carried out by people with reduced freedom of choice. However, against this it can be argued people who smoke might be unable to stop wanting to use, but whether to act on those desires remains under voluntary control [93]. If perceived as a voluntary response to an involuntary desire, invasive interventions toward people who smoke might be harder to justify.

### Non-paternalistic help to smokers with no desire to quit

It is difficult to imagine that authorities in western countries, in an endgame phase of the century-old cigarette epidemic, will give in to some people' wish to be able to enjoy their cigarettes in peace for further austerity measures. Although today’s users of cigarettes appear to be consonant and rational, and despite the fact that those who smoke have become an aging minority who, due to their lack of socio-economic capital, perhaps could have appealed to greater empathy from the authorities than what is the case, everything indicates that the regulatory and normative climate will harden for them.

In an even more hostile social, economic, and normative climate for smoking, people who are unwilling or unable to quit nicotine can have an escape route in non-combustible recreational nicotine products such as snus, nicotine pouches or e-cigarettes—as suggested by the Royal College of Physicians [94]. Already in 1976, the pioneering tobacco researcher Michael Russell wrote in the British Medical Journal *‘people smoke for the nicotine but die from the tar’* [95]. Nicotine in itself does not cause cancer and is assumed to play a minor role in most smoking-related diseases [94, 96]. Nicotine replacement therapies (patches, gums, lozenges, spray and inhalator) is generally regarded as safe and are approved for use in smoking cessation in most countries. They are moderately effective in experimental settings [96], but in real-world situations the effectiveness is low because the effect wears off [97, 98], but more importantly because these products only appeal to the minority with quit intentions and even in this group their use is small [99]. As compared to pharmaceutical nicotine products, the range of recreational nicotine products may have appeal as replacements for cigarettes in wider segments of people who smoke [96], also among those without quit intentions [100–102]. But realizing the potential benefits of recreational, non-combustible nicotine products will require politicians to implement risk-proportionate regulation regime that can nudge people who smoke to a product switch, in a soft paternalistic way—comparable to facilitating for choice of “healthier” food in cafeterias and in shops [14]. As Gostin and Gostin [41] have noted, “only the government can make such choices easier as it may mean altering the informational, built or socio-economic environment, which is beyond the ability of any single individual or group”.

However, such a pragmatic approach to reduce smoking and smoking-related disease will imply that any goal of a nicotine-free society must be abandoned and instead be replaced by goal of smoke-free society. Moreover, increasing attractiveness of these products for those who smoke—the at-risk population—can have the unintentional spillover effect of increased use among young people who have never smoked. In a risk-use equilibrium perspective of the net public health effect, a quite high number of people who have never smoked will have to start using these products to even out the benefit from each smoker who make the switch [103, 104]. The question for government regulators—as discussed from various ethical angles [105–109]—is how to strike the right balance between making potentially lower risk nicotine products accessible, sufficiently appealing and effective to displace smoking, while discouraging use by those who do not smoke, especially youth. Excessive regulation may perpetuate adult smoking. The two objectives can and should co-exist. However, the existence of this tradeoff may not be fully acknowledged within the tobacco control community [110].

### Paternalism in a smoke-free society

In Norway and Sweden, the overall prevalence of people who smoke daily is getting close to 5% and with very low annual rates of smoking initiation among youth (below 2%), we have finally started to envision a smoke-free society. A similar development is occurring in several other countries, e.g. New Zealand, As there will be steadily less people to cure for smoking dependency, the therapeutic function for recreational nicotine products in smoking cessation will eventually fade away. Then, we are left only with the values these products provide as pure consumer goods. In Norway, the authorities have started to regulate the non-combustible products quite strictly. Nicotine pouches and heated tobacco products have been banned and flavors in e-cigarettes will be restricted to tobacco. Apparently, regulators have set out to implement coercive paternalistic interventions toward recreational use of non-combustible nicotine that once proved to be functional in curbing smoking. The measures have been justified out of fears of an increase in future use among young people, based on a precautionary approach.

However, future use of these products will probably be low given that the proportion of those who smoke—up until now the largest reservoir of their potential users—will be reduced. Fewer future users, combined with the fact that these products are assumed to have low to moderate health hazards [96], will imply that they might not represent a large enough threat to public health to justify coercive paternalistic interventions.

On the other side, there is a possibility that nicotine use in the population will increase if these products come without the deterrence of harm. The effort to reduce nicotine use has been driven by the harms of smoking—not by opposition to the effects of nicotine as a drug. If nicotine can be provided at acceptably low risk within society’s normal risk appetites for consumption, it may activate an underlying demand that up until now has been suppressed by the harm from smoking [111]. But why would there be an underlying desire to use nicotine if it is not to cure smoking?

### Paternalism to suppress an underlying desire for nicotine

Nicotine has psychoactive effects that provide functional benefits and pleasurable sensations to its users. It has been found to improve certain cognitive functions, including attention, memory, and processing speed [112]. It can temporarily increase alertness and focus, making users feel more mentally sharp. Nicotine stimulates the release of neurotransmitters like dopamine, serotonin, and norepinephrine, which can help regulate mood. As a result, some users report feeling more relaxed, less anxious, or experiencing an improved sense of well-being [113]. Nicotine can act as an appetite suppressant, which may help some individuals control their food intake and support weight management efforts. Furthermore, research is ongoing, but there is some evidence to suggest that nicotine may have potential therapeutic benefits for conditions such as Alzheimer's disease, Parkinson's disease, and attention deficit hyperactivity disorder (ADHD) [114].^5^

Thus, nicotine seems to provide valuable benefits especially for people whose lives are difficult and stressful, those prone to anxiety or distraction or those who just enjoy the strange mixture of its stimulating and calming effects. Our concerted public health efforts to reduce disease and death caused by smoking could have deterred people who otherwise would have benefited from or enjoyed the mood-regulating and cognitive benefits of nicotine had it been available in safer forms.

Consequently, understanding nicotine use through the lenses of tobacco harm reduction might turn out to be incomplete for predictions of the direction and destination of the future consumer nicotine market. Tobacco harm reduction might become an interim stage in the evolution toward recreational use of non-combustible products that fall within the normal societal tolerance for risk. For this scenario to occur, it requires that the authorities are willing to put in place a communication strategy that accurately convey the harms and benefits of nicotine, so that current misperceptions [46, 115] will be corrected. So far, nothing points in that direction.

## Conclusion

If people who smoke are aware of and have accepted the risks, are willing to pay the price, smoke exclusively in designated areas, and make decisions uninfluenced by persuasive messages from manufacturers – a further tightening of anti-smoking measures may, from a legitimation perspective, appear challenging for regulators. Regulators must determine the line between an ethically justified interference with the freedom of individuals who smoke and the exaggerated infringement of their freedom, disproportionate to their right to live as they choose. In countries with an already advanced infrastructure for tobacco control, this dilemma might become quite intrusive for regulators, especially if a sizable fraction of the remaining group of people who smoke has little or no wish to quit smoking.

We recommend that a further intensification of smoking control in countries that already have a well-developed policy in this area requires that regulators start to exploit the opportunity that lies in the ongoing diversification of the recreational nicotine market. Providing people who smoke with an escape route to combustion-free nicotine products with reduced risk should be a naturel part of a humane tobacco control policy.

## Data Availability

Not applicable.

## Abbreviations

US: United States
DSMS-5: Diagnostic and Statistical Manual of Mental Disorders
ADHD: Attention Deficit Hyperactivity Disorder

## Footnotes

1 "Consonant" smokers are described as holding relatively positive attitudes about smoking and not expressing a wish to stop, whereas "dissonant" smokers are described as continuing to smoke despite of a wish to stop [7]. Both groups should benefit from alternative forms of nicotine intake.

2 Here, the response from the tobacco control community has been that, for example, tax increases must nevertheless be considered socially progressive—and not regressive—because the reduction in demand has proven to be greatest in the groups where smoking is most widespread and consequently narrow the social disparities in future health status [35].

3 This reasoning is used, for instance, in support of coercive treatment of drug addicts [36].

4 A lung cancer patient sought compensation from a tobacco manufacturer. The case was litigated in three instances, and all ended in loss. In the court rulings, emphasis was placed on the fact that people who smoke must have been informed of the health risks and therefore had been giving informed consent.

5 See complete list of studies on beneficial effects from nicotine here: https://safernicotine.wiki/mediawiki/index.php/Nicotine_therapeutic_benefits
